# Notch-1 knockdown suppresses proliferation, migration and metastasis of salivary adenoid cystic carcinoma cells

**DOI:** 10.1186/s12967-015-0520-2

**Published:** 2015-05-20

**Authors:** Wei Chen, Gang Cao, Xinran Yuan, Xiang Zhang, Qingqing Zhang, Yinglan Zhu, Zhen Dong, Senlin Zhang

**Affiliations:** Department of Stomatology, Jinling Hospital, School of Medicine, Nanjing University, 305 East Zhongshan Road, 210002 Nanjing, Jiangsu China; Department of Immunology & Rheumatology, Drum Tower Hospital, School of Medicine, Nanjing University, Nanjing, Jiangsu China

**Keywords:** Notch-1, RNA interference, Perineural invasion, Metastasis, Adenoid cystic carcinoma

## Abstract

**Background:**

Notch-1 promotes invasion and metastasis of cancer cells but its role in salivary adenoid cystic carcinoma (SACC) remains unelucidated. Here, we sought to investigate the effect of Notch-1 knockdown on the invasion and metastasis of SACC cells.

**Methods:**

Stable ACC-M cells whose Notch-1 was silenced by lentiviral vectors were established. Cellular proliferation was evaluated by the MTT assays and clonogenic assays, apoptosis by flow cytometry and the migration of ACC-M cells by Transwell assays. Metastasis was evaluated by examining the number of lung nodules in Balb⁄c nu⁄nu nude mice bearing subcutaneous SACC xenografts.

**Results:**

Our MTT assay revealed that Notch-1 knockdown significantly suppressed the proliferation of ACC-M cells compared with non-infected or scrambled control cells. Clonogenic assays further showed that Notch-1 knockdown significantly suppressed the clonogenic growth of ACC-M cells (*p* < 0.01 vs. controls). Our flow cytometry demonstrated that Notch-1 knockdown was associated with a significantly higher proportion of late apoptotic and necrotic cells (*p* < 0.01 vs. controls). Transwell assays revealed that Notch-1 knockdown markedly reduced the migratory capacity of ACC-M cells (*p* < 0.01 vs. controls) and xenograft studies showed that the number of metastatic nodules in the lung surface was significantly lower in nude mice bearing xenografts with Notch-1 knockdown compared to those bearing control xenografts (*p* < 0.01 vs. controls).

**Conclusion:**

Notch-1 knockdown suppresses the growth and migration of SACC cells *in vitro* and the metastasis of SACC cells *in vivo.* Notch-1 may be a new candidate target in SACC.

## Background

Salivary adenoid cystic carcinoma (SACC) accounts for approximately 7.5 % of all salivary gland epithelial malignancies and 4 % of all salivary gland neoplasms [1]. It is characterized by heterogeneous phenotypic features and persistently progressive biological behavior. SACC patients have a dismal long-term outcome: in a cohort of 160 SACC patients, the overall survival stood at 89 % at 5 years, but declined to 40 % at 15 years [2]. Major contributors to the poor long-term prognosis of SACC patients are local recurrence associated with perineural invasion (PNI) and distant hematogenous metastasis, particularly to the lungs [3,4].

The Notch signaling pathway is a highly evolutionarily conserved signaling pathway that regulates various physiological processes, including stem cell maintenance, differentiation, proliferation and apoptosis [5,6]. Recently, accumulating evidence has implicated aberrant Notch signaling in the oncogenesis of many human malignancies. However, little is known about the differential role of various components of the Notch pathway in SACC. Notch-1, one of the key receptors in the Notch signaling pathway, encodes an important member of the Notch family proteins [7]. Whole genome sequencing of adenoid cystic carcinoma cells has identified mutations in Notch-1 though the functional significance of the mutations is unknown [8]. Additionally, Notch-1 expression was found to be elevated in SACC tissues [9]. Notch-1 has also been shown to promote the proliferation of SACC and head and neck cancer cells [9,10]. We have demonstrated by microarray analysis that Notch-1 and Notch-4 are implicated in the pathobiology of SACC by influencing perineural invasion [11]. Furthermore, Notch-1 cross-talk has also been reported with other major cell growth and apoptotic regulatory pathways through modulating the activity of transcription factors such as nuclear factor-κB (NF-κB) and Wnt/β-catenin signaling [12,13].

Notch-1 was shown to promote the invasion and metastasis of esophageal carcinoma cells by inducing epithelial-mesenchymal transition (EMT) [14]. Activation of Notch-1 signaling was demonstrated in gefitinib-resistant lung cancer cells undergoing EMT [15]. However, a role of Notch-1 has not been suspected in the invasion and metastasis of SACC cells. We hypothesized that Notch-1 may be involved in the pathogenesis of SACC by promoting the invasion and metastasis of SACC cells. In the current study, we sought to elucidate the role of Notch-1 in invasion and metastasis of SACC cells by examining the proliferation and migration of SACC cells whose Notch-1 expression was knocked down by lentiviral vector-mediated RNA interference (RNAi), and by evaluating the presence of metastatic nodules in nude mice bearing SACC xenografts.

## Materials and methods

### Cell lines and cell culture condition

ACC-M and 293 T cells were purchased from the Cell Bank for Type Culture Collection, Chinese Academy of Sciences (Beijing, China). The cells were cultured in Dulbecco’s modified Eagle’s medium (DMEM) (Invitrogen. Carlsbad, CA, USA) supplemented with 10 % of fetal bovine serum (FBS) (Gibco, Invitrogen), 2.05 mM L-glutamine, 100 μg/mL of penicillin and 100 μg/mL of streptomycin at 37 °C with 5 % CO_2_.

### Generation of stable Notch-1-silencing cells

**Fig. 1:**
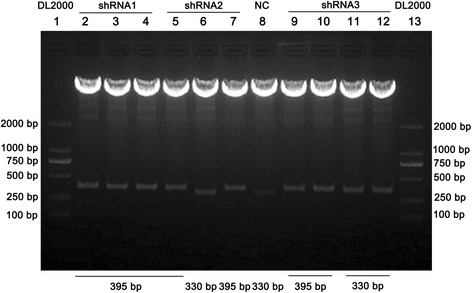
Validation results of recombinant vector by *Kpn*Iand *EcoR*Idouble digestion. Lanes *1* and *13* were Marker DL2000. Lanes *2*, *3* and *4* were three clones of shRNA1, which were all confirmed as positive clones (395 bp). Lanes *5*, *6* and *7* were three clones of shRNA2, and lane *5* and *7* were confirmed as positive clones (395 bp). Lanes *9*, *10* and *11* were three clones of shRNA3, and lane *9* and *10* were confirmed as positive clones (395 bp). Lane *8* was a negative control (330 bp)

Lentiviral vectors (pLenR-GPH, pMDLg/pRRE, pRSV-Rev, and pMD2.G) were purchased from Innovation Biotechnology (Shanghai, China). The sequences of siRNAs are shown in Table 1. SiRNAs were designed according to the cDNA sequence of the Notch-1 gene (NM_017617.3) in GenBank and were inserted into the *BamH*I and *EcoR*I site of linearized pLenR-GPH according to the manufacturer’s instructions. The recombinant vectors were confirmed by endonuclease restriction analysis with *Kpn*I and *EcoR*I and sequencing and were designated pLenR-Notch-1-shRNA1, 2, and 3 (Fig. 1).

**Table 1:** Oligonucleotide sequences of *Notch-1* specific shRNAs

Name	Base sequence
shRNA1-F	5′-GATCC*CTTTGTTTCAGGTTCAGTATT*CTTCCTGTCAGA*AATACTGAACCTGAAACAAAG*TTTTTG-3′
shRNA1-R	5′-AATTCAAAAA*CTTTGTTTCAGGTTCAGTATT*TCTGACAGGAAG*AATACTGAACCTGAAACAAAG*G-3′
shRNA2-F	5′-GATCC*CGCTGCCTGGACAAGATCAAT*CTTCCTGTCAGA*ATTGATCTTGTCCAGGCAGCG*TTTTTG-3′
shRNA2-R	5′-AATTCAAAAA*CGCTGCCTGGACAAGATCAAT*TCTGACAGGAAG*ATTGATCTTGTCCAGGCAGCG*G-3′
shRNA3-F	5′-GATCC*CCGGGACATCACGGATCATAT*CTTCCTGTCAGA*ATATGATCCGTGATGTCCCGG*TTTTTG-3′
shRNA3-R	5′-AATTCAAAAA*CCGGGACATCACGGATCATAT*TCTGACAGGAAG*ATATGATCCGTGATGTCCCGG*G-3′
NC-F	5′-GATCC*TTCTCCGAACGTGTCACGT*CTTCCTGTCAGA*ACGTGACACGTTCGGAGAA*TTTTTG -3′
NC-R	5′-AATTCAAAAA*TTCTCCGAACGTGTCACGT*TCTGACAGGAAG*CGTGACACGTTCGGAGAA*G -3′

Annotation: shRNA1-3 indicates the oligonucleotide sequence of *Notch-1*. NC, negative control. The sequences in italics represent the forward (F) and reverse (R) target sequence

**Fig. 2:**
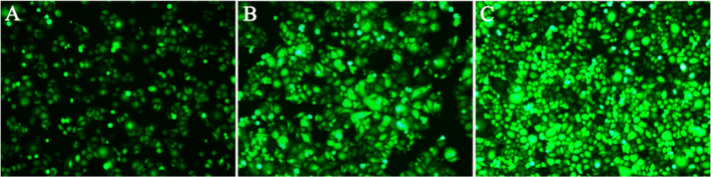
The representative examples showed stable transfectants procured by different quantity of purified lentivirus. **a** The ACC-M cells were stable transfected by 0.01 μl purified lentivirus. **b** The ACC-M cells were stable transfected by 0.1 μl purified lentivirus. **c** The ACC-M cells were stable transfected by 1 μl purified lentivirus. The cell transfection rate was directly observed by green fluorescent protein (GFP) positive, which reached to 90 %. (Original magnification: ×100)

ACC-M cells were seeded at a density of 2 × 10^5^⁄well in 6-well plates. After 24 h incubation, the cells were transfected with appropriate lentivirus vectors at a multiplicity of infection of 10 as instructed by the manufacturer (Innovation Biotechnology). Stable ACC-M cells containing the lentivirus vectors were established after selections with appropriate antibiotics. Fluorescent microscopy revealed a transfection rate of 90 % (Fig. 2).

### Quantitative RT-PCR

Total cellular RNA was extracted using TRIzol reagent as instructed by the manufacturer (Invitrogen). First strand cDNA synthesis was made using 1 μ*g* total cellular RNA with a commercially available kit (Qiagen, Valencia, CA, USA). Quantitative RT-PCR was performed with QuantiTect SYBR Green PCR master mix (Qiagen) using the Rotor-Gene RG-3000 Real-Time Thermal Cycler (Corbett Research, Sydney, Australia) and Rotor-Gene software version 6.0. The PCR was carried out in a 20 μL reaction containing 10 μL SYBR-Green master mix, 0.5 μL each primer, and 0.5 μL cDNA template. The primer sequences were as follows: *Notch-1*, 5′-AGTACTGTACCGAGGATGTGGACG-3′ (sense), and 5′-GAAGGAGGCCACACGGTCAT-3′ (antisense) and *GAPDH*, 5′-GGAGTCCACTGGCGTCTTC-3′ (sense) and 5′-GCTGATGATCTTGAGGCTGTTG-3′ (antisense). The PCR was run in triplicate at 95 °C for 2 min followed by 45 cycles of 95 °C for 15 s, 58 °C for 20 s, and 72 °C for 20 s. Comparative quantification was done using the 2^-⊿⊿Ct^ method. Each sample was performed in triplicate.

### Western blotting assays

Cells were washed twice with cold phosphate-buffered saline and lysed on ice in buffer containing protease inhibitors. Equal amounts of protein (20 μ*g*/lane) from the cell lysates were electrophoresed on 10 % acrylamide gels. After SDS-PAGE, proteins were transferred to a polyvinylidene difluoride membrane. The membrane was incubated for 2 h in PBS plus 0.1 % Tween 20 and 5 % non-fat skim milk to block non-specific binding. Subsequently, the membrane was incubated for 2 h with an antibody against Notch-1 (Abcam, Cambridge, UK) and an anti-GAPDH antibody. The protein bands were visualized using an ECL detection kit (Amersham Pharmacia Biotech, Piscataway, NJ, USA). Notch-1 expression was normalized against GAPDH. The experiment was repeated three times.

### Cell proliferation and clonogenic assays

For cell proliferation studies, cells were seeded at a density of 1 × 10^3^ per well on 96-well plates. After 8 days, cell viability was assessed by the tetrazolium-based semi-automated colorimetric 3-[4, 5-dimethylthiazol-2-yl] -2, 5-diphenyltetrazolium bromide (MTT) reduction assays. Spectrometric absorbance was read at 490 nm using a microplate reader. For clonogenic survival assays, cells were plated at 400 cells/well in 6-well culture dishes 96 h post lentiviral infection. After 1 week, cell colonies were fixed with 4 % paraformaldehyde and stained with crystal violet (Sigma). An aggregate of at least 50 cells was considered a colony and was counted. Each group was repeated three times.

### Flow cytometry

Stable Notch-1 knockdown ACC-M cells were washed once with PBS, trypsinized and washed again in PBS with 2 % FBS and fixed in 70 % ice-cold ethanol for 2 h. Cells were washed, and stained with propidium iodide (30 μg/mL) and treated with RNase (1 mg/mL) in PBS plus 0.5 % (v/v) Tween 20 and 2 % FBS. Stained cells were analyzed on a flow cytometer (Beckman Coulter, Inc., Fullerton, CA, USA). The proliferation index value was defined as the total number of cells in the S and G_2_/M phases divided the total number of cells. Furthermore, apoptotic cells were detected by staining with propidium iodide and annexin V. Briefly, cells were incubated with 1 mg/mL annexin V-fluorescein isothiocyanate and 1 mg/mL propidium iodide for 15 min in the dark at 4 °C. The number of stained cells was analyzed by a Beckman Coulter flow cytometer. ModFit program (Verity Software House Inc., USA) was used to analyze the cell-cycle profiles and the proportion of apoptotic cells.

### Cell migration assays

Cell migration was assessed in a modified Transwell Boyden chamber (Costar), in which the two chambers were separated by a polycarbonate membrane (pore diameter, 8.0 μm) (Millipore Corp., Billerica, MA, USA). Transwell chambers were coated on the upper surface with Matrigel basement membrane (BD Biosciences, San Diego, CA, USA). Cell suspensions containing 1 × 10^5^ cells in DMEM supplemented with 1 % FBS were added to the upper chamber. Cells were incubated for 12 h at 37 °C with 5 % CO_2_. At the end of the incubation, the cells on the upper surface of the filter were completely removed by wiping with a cotton swab. The filters were then fixed in methanol and stained with hematoxylin and eosin (H&E). Cells that had invaded the Matrigel and had reached the lower surface of the filter were counted under a light microscope at a magnification of × 400. We chose five visual fields and counted the number of migrated cells on the lower surface of the filter.

### Xenograft studies

Six- to 8-week-old Balb⁄c nu⁄nu nude mice were inoculated with 2 × 10^5^ ACC-M cells in 200 μL culture medium via the tail vein. Six weeks after inoculation, all animals were euthanized and the lungs were removed. The number of metastatic nodules on the surface of the lungs was counted. Then, the metastatic nodules were paraffin-embedded for immunohistochemical staining. The study protocol was approved by the local animal ethics committee of Jinling Hospital (Project NO.2014GJJ-003) and was carried out in accordance with the local and state regulations regarding the use of animals for experimental purposes.

### Immunohistochemistry

An S-P Kit (Gene Tech, Shanghai) was used for immunohistochemical analysis. In brief, paraffin sections (4 μm thick) were deparaffinized in xylene and rehydrated in gradient alcohol. Antigen retrieval was performed by microwave treatment in 0.01 mol/L sodium citrate buffer (pH = 6.0) for 15 min, cooled to room temperature for 1 h, and then washed with phosphate-buffered saline (PBS). Endogenous peroxidase activity was inactivated with 0.3 % hydrogen peroxide for 30 min and non-specific blocked with avidin/biotin blocking solutions and 10 % normal goat serum. The sections were then incubated overnight with primary antibody against Notch-1 (1:150, Abcam, Cambridge, UK). Biotinylated goat anti-rabbit IgG were used as secondary antibody. The immunoreactions were detected by staining with 3,3′-diaminobenzidine. Negative controls were performed by omitting the primary antibody. The slides were evaluated in a double-blinded manner by three pathologists independently, and the scores were averaged. The proportion of positive tumor cells was recorded according to the following classification: 0, no cells stained; 1, <1/3 of cells stained; 2, 1/3–2/3 of cells stained; and 3, >2/3 of cells stained. The intensity of the coloring was recorded according to the following classification: 0, no coloring; 1, stramineous; 2, buffy; and 3, dark brown. The two scores were combined to obtain the final one: scores equal to 0 indicated negative (−), 1 indicated slightly positive (+), 2–4 indicated moderately positive (++), and 5–6 indicated strongly positive (+++).

### Statistical analysis

Data were expressed as mean ± standard deviation (SD) and analyzed using SPSS 17.0 statistical software (SPSS Inc., Chicago, IL, USA). Data were tested for statistical significance using analysis of variance (ANOVA) or two-tailed unpaired Student’s *t*-test. Normally distributed, continuous variables were compared using one-way ANOVA. When ANOVA produced a significant difference between groups, multiple comparisons of group means were performed using the Bonferroni procedure with a type I error adjustment. All *p* values were two-sided, and significance was defined as *p* < 0.05.

## Results

### Efficiency of Notch-1 knockdown by lentiviral vectors

**Fig. 3:**
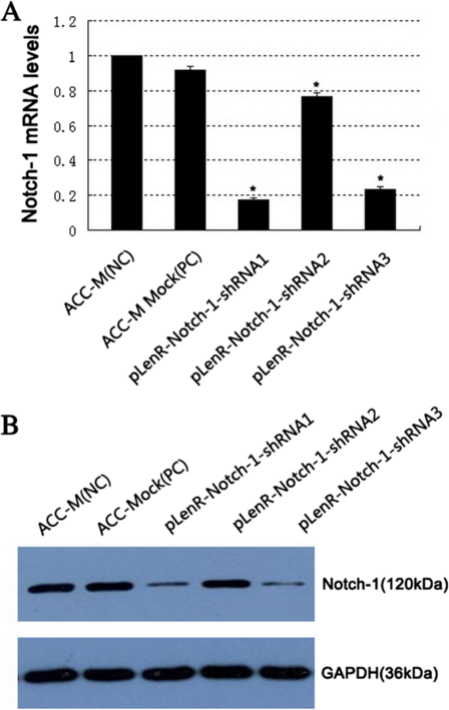
Efficiency of Notch-1 knockdown in ACC-M cells by lentiviral vectors bearing shRNA. ACC-M cells were infected with lentiviral vectors pLenR-Notch-1-shRNA1, 2, and 3 targeting Notch-1 or scrambled shRNA vector (ACC-Mock (PC)) as detailed in Materials and methods. The Notch-1 mRNA transcript levels were determined by quantitative RT-PCR **(a)** and Notch-1 protein expression by Western blotting assays **(b)**. Data shown in **(c)** are expressed as mean ± SD of at least three independent experiments in triplicate. Representative blots are shown in **(d)** of at least three independent experiments. Notch-1 protein expression was normalized against GAPDH. *p < 0.01 compared with non-infected or scrambled control ACC-M cells

We infected ACC-M cells with a panel of lentiviral vectors bearing siRNA targeting Notch-1 and quantitative RT-PCR revealed that all three lentiviral vectors caused significant reduction in the mRNA transcript levels of Notch-1 (*p* < 0.01 vs. non-infected ACC-M cells (negative control) or ACC-M cells infected with lentiviral vectors bearing scrambled siRNA (positive control, ACC-M Mock)) (Fig. 3a). Furthermore, pLenR-Notch*-*1-shRNA1 caused the most significant inhibition in Notch-1 mRNA transcript levels. Consistently, immunoblotting assays showed marked reduction in the protein level of Notch-1 by the lentiviral vectors, most noticeably pLenR-Notch-1-shRNA1 (Fig. 3b), which was used for subsequent experiments.

### Notch-1 knockdown suppresses clonogenic growth of SACC cells in vitro

**Fig. 4:**
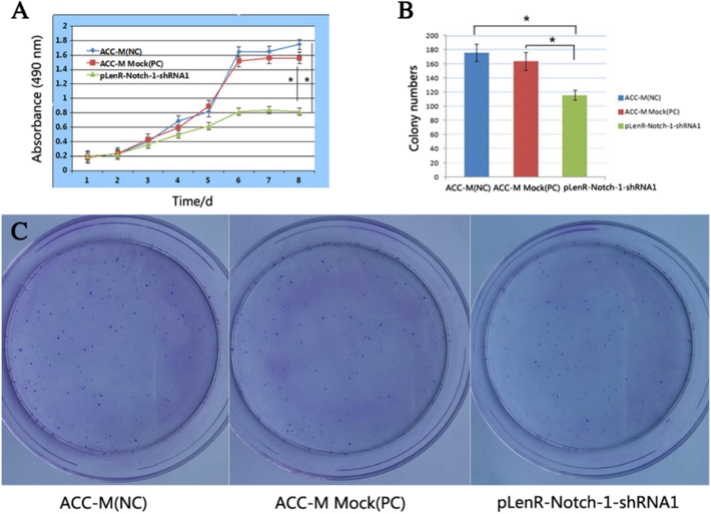
Notch-1 knockdown suppresses clonogenic growth of salivary adenoid cystic carcinoma cells in vitro. Cells were treated as in described in Materials and methods. Cellular proliferation was evaluated using the MTT assays **(a)** and colony formation was examined by clonogenic assays **(b, c)**. Data shown in **(a)** and **(b)** are expressed as mean ± SD of at least three independent experiments in triplicate. *p_1, 3_ < 0.01, *p*_2, 3_ < 0.01. (*1*, ACC-M (NC); *2*, ACC-M Mock (PC); *3*, pLenR-Notch-1-shRNA1)

To examine whether lentiviral vector-mediated knockdown of Notch-1 impacted on the proliferation of cancer cells, we examined the growth of lentivirally infected ACC-M cells by MTT assays. We found that Notch-1 knockdown by pLenR-Notch-1-shRNA1 significantly suppressed the proliferation of ACC-M cells compared with non-infected ACC-M cells or ACC-M cells infected with lentiviral vectors bearing scrambled siRNA cells (*p* < 0.01) (Fig. 4a). Clonogenic assays further showed that Notch-1 knockdown significantly suppressed the clonogenic growth of ACC-M cells (116 ± 7) compared with non-infected ACC-M cells (176 ± 13) or ACC-M cells infected with lentiviral vectors bearing scrambled siRNA (164 ± 12) (*p* < 0.01) (Fig. 4b, c). These findings demonstrated that Notch-1 knockdown significantly suppressed the clonogenic growth of salivary adenoid cystic carcinoma cells *in vitro*.

### Notch-1 knockdown induced late apoptotic and necrotic death of SACC cells in vitro

**Fig. 5:**
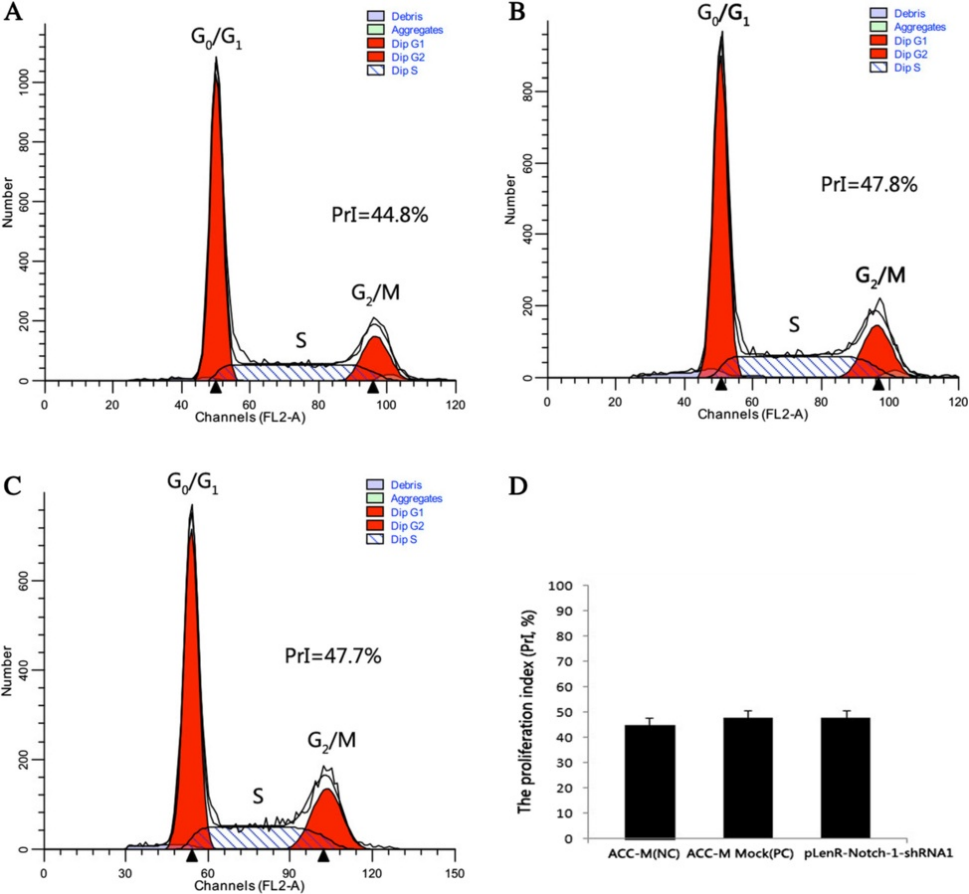
Notch-1 downregulation did not impact on the cell cycle distribution of salivary adenoid cystic carcinoma cells in vitro. Cells were treated as in described in Materials and methods. No significant decreases in PrI were found in the pLenR-Notch-1-shRNA1 stably transfected cells compared with negative or positive control cells (panel **d**, *P*_A,C_ > 0.05, *P*_B,C_ > 0.05). Statistical analysis was performed with one-way ANOVA. (PrI, proliferation index value) **(a)** ACC-M (NC); **(b)** ACC-M Mock (PC); **(c)** pLenR-Notch-1-shRNA1

We first investigated whether the growth-inhibitory effect of Notch-1 knockdown on ACC-M cells was due to reduced DNA synthesis. Our flow cytometric analysis revealed no statistically significant difference in the proliferation index of Notch-1 knocked down cells, non-infected ACC-M cells or ACC-M cells infected with lentiviral vectors bearing scrambled siRNA (*p* > 0.05) (Table 2, Fig. 5), indicating that Notch-1 downregulation did not impact on the cell cycle distribution of ACC-M cells.

**Table 2:** Cell cycle distribution of ACC-M cells

Group	G_0_/G_1_ (%)	S (%)	G_2_/M (%)	Proliferation index (%)
ACC-M (NC)	55.2 ± 3.01	28.8 ± 1.19	16.0 ± 1.34	44.8 ± 3.01
ACC-M Mock (PC)	52.2 ± 3.14	30.7 ± 0.75	17.0 ± 2.16	47.8 ± 2.74
pLenR-Notch-1-shRNA1	52.3 ± 2.71	27.7 ± 3.26	20.0 ± 1.50	47.7 ± 2.95

*P*_1,3_ > 0.05, *P*_2,3_ > 0.05, *P*_1,2_ > 0.05. 1, ACC-M (NC); 2, ACC-M Mock (PC); 3, pLenR-Notch-1-shRNA1

**Fig. 6:**
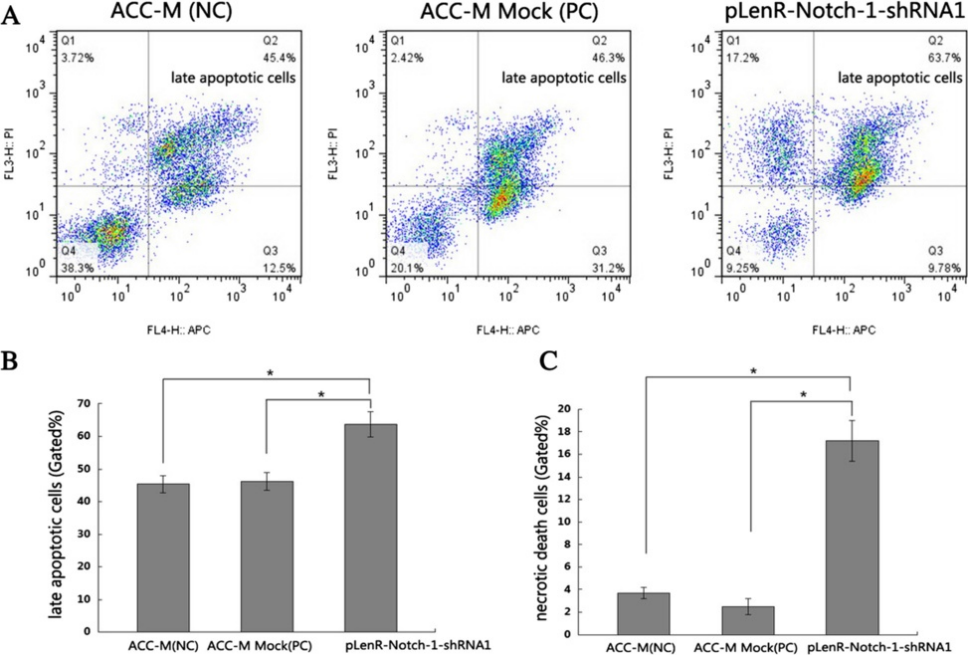
Notch-1 knockdown induced late apoptotic and necrotic death of salivary adenoid cystic carcinoma cells in vitro. Cells were treated as in described in Materials and methods and were stained by annexin V and propidium iodide. Cell death was examined by flow cytometry (panel **a**). Data shown in **(b)** and **(c)** are expressed as mean ± SD of at least three independent experiments in triplicate. *p_1, 3_ < 0.01, *p*_2, 3_ < 0.01. (*1*, ACC-M (NC); *2*, ACC-M Mock (PC); *3*, pLenR-Notch-1-shRNA1)

We next investigated whether the growth-inhibitory effect of *Notch-1* knockdown was associated with changes with apoptotic or necrotic death of lentivirally infected ACC-M cells. Our flow cytometry showed that Notch-1 knockdown was associated with a significantly higher proportion of late apoptotic cells (63.7 ± 3.8 %) compared to non-infected ACC-M cells (45.4 ± 2.6 %) or ACC-M cells infected with lentiviral vectors bearing scrambled siRNA (46.3 ± 2.7 %) (*p* < 0.01) (Fig. 6a and b). Furthermore, Notch-1 knockdown was also associated with a significantly higher proportion of necrotic cells (17.2 ± 1.8 %) compared to non-infected ACC-M cells (3.7 ± 0.5 %) or ACC-M cells infected with lentiviral vectors bearing scrambled siRNA (2.4 ± 0.7 %) (*p* < 0.01) (Fig. 6c). We also noticed that lentiviral vectors bearing scrambled siRNA caused a noticeable increase in the proportion of early apoptotic cells (31.2 ± 1.4 %) vs. Notch-1 knockdown (9.8 ± 0.8 %; *p* < 0.01). These results suggest that knockdown of Notch-1 caused a significant increase in the proportion of late apoptotic and necrotic ACC-M cells.

### Notch-1 knockdown inhibits the migratory capacity of SACC cells

**Fig. 7:**
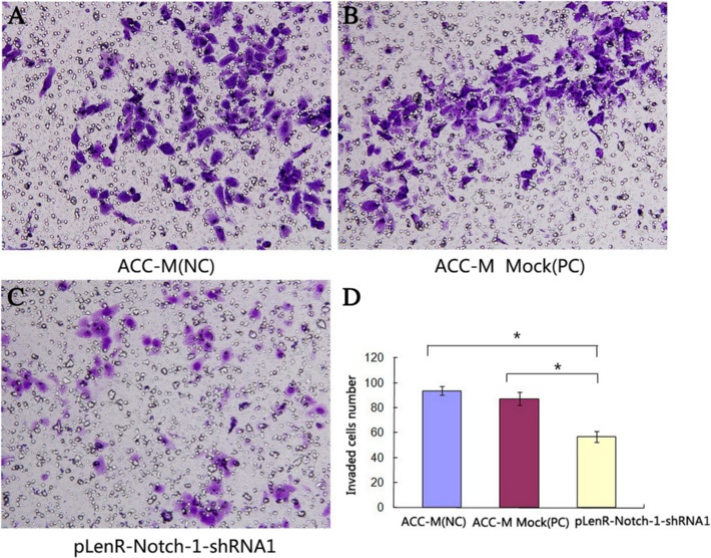
Notch-1 knockdown inhibits the migratory capacity of salivary adenoid cystic carcinoma cells. Cells were treated as in described in Materials and methods. The migration of cells was assessed by Transwell assays. Data shown in **(d)** are expressed as mean ± SD of at least three independent experiments in triplicate. The average invaded cells number of the groups ACC-M (NC), ACC-M Mock (PC) and pLenR-Notch-1-shRNA1 were (93.7 ± 3.5), (87.3 ± 5.1) and (56.7 ± 4.2), respectively. Representative photographs are shown in **(a** to **c)** of at least three independent experiments. *p_1, 3_ < 0.01, *p*_2, 3_ < 0.01. (*1*, ACC-M (NC); *2*, ACC-M Mock (PC); *3*, pLenR-Notch-1-shRNA1)

**Fig. 8:**
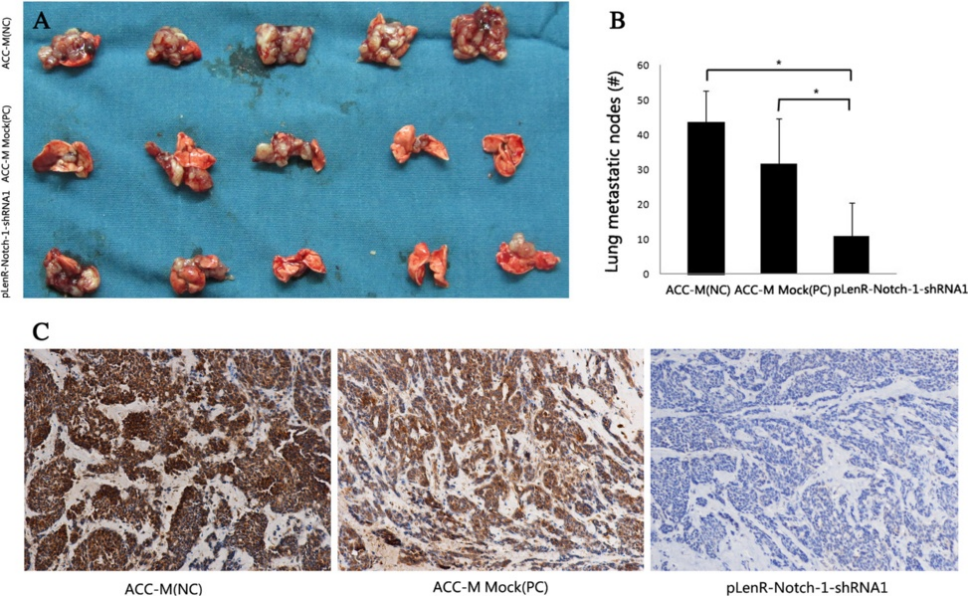
Notch-1 knockdown inhibits the migratory capacity of salivary adenoid cystic carcinoma cells. Mouse xenografts bearing SACC cells were established as detailed in Materials and methods. Tissue sections were made of xenografts after 6 weeks and immunohistochemical staining was done using anti-Notch-1 antibodies **(a)**. Photographs of gross metastatic lung nodules is shown in **(b)** and the mean number of lung metastatic nodules is shown in **(c)** *p_1, 3_ < 0.01, *p*_2, 3_ < 0.01. (*1*, ACC-M (NC); *2*, ACC-M Mock (PC); *3*, pLenR-Notch-1-shRNA1)

Notch-1 has been implicated in tumor metastasis in a variety of cancer types [14]. We investigated whether Notch-1 downregulation influenced the migratory capacity of salivary adenoid cystic carcinoma cells. Our Transwell assays showed that ACC-M cells infected with pLenR-Notch-1-shRNA1 exhibited a markedly reduced migratory capacity compared with non-infected ACC-M cells or ACC-M cells infected with lentiviral vectors bearing scrambled siRNA (*p* < 0.01) (Fig. 7). We then inoculated nude mice with lentivirally infected ACC-M cells and evaluated the presence of metastatic nodes in the lungs after 6 weeks. Immunohistochemical staining of tumor xenografts showed a marked reduction in the expression of Notch-1 in ACC-M cells whose Notch-1 was knocked down by pLenR-Notch-1-shRNA1 compared to non-infected ACC-M cells or ACC-M cells infected with lentiviral vectors bearing scrambled siRNA (immunoreactivity score: Notch-1 knockdown, 0.0 vs. scrambled siRNA, 5.0; *p* < 0.01) (Fig. 8c). Furthermore, the number of metastatic nodules in the lung surface was significantly lower in nude mice bearing xenografts with Notch-1 knockdown compared to those bearing control xenografts (Fig. 8a and b). The results indicated that Notch-1 was also implicated in the metastasis of salivary adenoid cystic carcinoma cells *in vivo.*

## Discussion

Notch signaling regulates cell fate, stem cell maintenance and the initiation of differentiation in many tissues and aberrant Notch signaling is intimately implicated in oncogenesis and development. Notch-1 expression was found to be elevated in SACC tissues [9], but its role in the growth and metastasis of SACC remains largely undefined. In the current study, we demonstrated that Notch-1 downregulation significantly suppresses the clonogenic growth and the migratory capacity of SACC cells *in vitro.* More importantly, Notch-1 knockdown markedly inhibited the formation of metastatic lung nodules in a mouse model bearing SACC xenograft. Our study has provided the piece of experimental evidence that Notch-1 is implicated in the growth and invasion of SACC *in vitro* and *in vivo*, paving the way for further elucidation of the role of Notch-1 in the oncogenesis and development of SACC.

We further demonstrated that the growth inhibitory effect of Notch-1 knockdown was at least partially associated with significantly enhanced late apoptosis and necrotic death of SACC cells. This is consistent with the findings by Ye *et al.* who demonstrated that Notch-1 knockdown promoted docetaxel induced growth inhibition and apoptosis of prostate cancer cells *in vitro* [16]. Li *et al.* also found that Notch-1 activation inhibited apoptosis of MDA-MB-231 breast cancer cells [17]. We further demonstrated that the growth inhibitory effect of Notch-1 knockdown was not due to altered cell cycle distributions of SACC cells. Our findings and those by other investigators suggest that Notch-1 regulates cancer cell growth at least partially by modulating apoptosis of these cancer cells.

Our previous microarray study revealed that Notch-1 and Notch-4 were notably overexpressed in the perineural invasion group, providing the hint of the involvement of Notch-1 in the invasion and metastasis of SACC [11,18]. Notch-1 was actively involved in promoting the metastasis of esophageal carcinoma cells by inducing EMT [14] and its activation was also implicated in potentiating EMT of gefitinib-resistant lung cancer cells [15]. The current study demonstrated that Notch-1 knockdown significantly inhibits the migratory capacity of salivary adenoid cystic carcinoma cells and reduces the number of metastatic nodules in the lung surface of mice bearing SACC xenografts, indicating that Notch-1 is implicated in the metastasis of salivary adenoid cystic carcinoma cells *in vivo.* Tumor progression is the process by which tumor cells acquire malignant properties, such as aggressive growth, perineural invasion, and metastasis. By targeting critical signaling molecules in the acquisition of malignant phenotypes of SACC, we may slow down the progression of SACC. Our results indicate that Notch-1 is implicated in the invasion and metastasis of SACC cells, and may be manipulated therapeutically to delay the migration and metastasis of SACC cells. Our findings of Notch-1 as a critical signaling molecule in SACC would lead to additional studies to delineate the role of Notch-1 in the oncogenesis and development of SACC and the underlying mechanism.

## Conclusions

In conclusion, we demonstrate that Notch-1 knockdown suppresses the growth and migration of SACC cells *in vitro* and the metastasis of SACC cells *in vivo*. As a key regulator of growth and metastasis of SACC cells, Notch-1 may be a new candidate target for the treatment of SACC.

## Abbreviations

SACC: Salivary adenoid cystic carcinoma
PNI: Perineural invasion
siRNA: Small interfering RNA
shRNA: Short hairpin RNA
NF-κB: Nuclear factor-κB
